# Mendelian Randomization Evidence for Gerotherapeutics

**DOI:** 10.1093/geroni/igaf122.1579

**Published:** 2025-12-31

**Authors:** Chia-Ling Kuo

**Affiliations:** UConn Health, Farmington, Connecticut, United States

## Abstract

Aging-related diseases and functional decline present major public health challenges. Mendelian randomization (MR) offers a robust approach for evaluating gerotherapeutic candidates by leveraging genetic variants as instrumental variables for drug exposures to infer their causal effects on aging outcomes. Additionally, the increasing availability of genetic instruments from genome-wide association studies (GWAS) has enhanced the ability to conduct rigorous MR analyses in this field. Successful MR studies require well-defined drug exposure proxies and careful selection of genetic instruments, such as biomarker levels or target gene expression. However, challenges remain, including limited knowledge of target genes, the restriction of protein GWAS to blood samples, and the lack of genetic instruments for RNA expression in highly expressed tissues. To ensure valid MR analyses, several statistical considerations must be addressed to uphold key MR assumptions. Strong genetic instruments are crucial for robust causal inference. Horizontal pleiotropy should be minimized to avoid bias, and sample overlap bias must be assessed to prevent inflated estimates. Finally, consistency across multiple MR methods strengthens the reliability of causal inferences. This presentation aims to provide a framework to assess MR studies on candidate gerotherapeutics by using genetic instruments linked to drug exposures to evaluate their effects on lifespan, healthspan, and aging-related traits. Systematically assessing the evidence for gerotherapeutic candidates is critical to identifying promising interventions, refining drug development strategies, and advancing the field of geroscience toward effective and targeted therapies for aging-related diseases.

